# Evaluation of Inertial Sensor Data by a Comparison with Optical Motion Capture Data of Guitar Strumming Gestures

**DOI:** 10.3390/s20195722

**Published:** 2020-10-08

**Authors:** Sérgio Freire, Geise Santos, Augusto Armondes, Eduardo A. L. Meneses, Marcelo M. Wanderley

**Affiliations:** 1 Laboratory for Performance with Interactive Systems (LaPIS), Federal University of Minas Gerais (UFMG), Belo Horizonte 31270-901, Brazil; augusto_armondes@hotmail.com; 2 Reasoning for Complex Data Lab (RECOD), Institute of Computing (IC), University of Campinas (UNICAMP), Campinas 13083-852, Brazil; geise.santos@ic.unicamp.br; 3 Input Devices and Music Interaction Laboratory (IDMIL)/Centre for Interdisciplinary Research in Music Media and Technology (CIRMMT), McGill University, Montreal, QC H3A 0G4, Canada; eduardo.meneses@mail.mcgill.ca (E.A.L.M.); marcelo.wanderley@mcgill.ca (M.M.W.)

**Keywords:** motion capture, MetaMotionR, sensors for music, Qualisys, guitar strumming, interactive music

## Abstract

Computing technologies have opened up a myriad of possibilities for expanding the sonic capabilities of acoustic musical instruments. Musicians nowadays employ a variety of rather inexpensive, wireless sensor-based systems to obtain refined control of interactive musical performances in actual musical situations like live music concerts. It is essential though to clearly understand the capabilities and limitations of such acquisition systems and their potential influence on high-level control of musical processes. In this study, we evaluate one such system composed of an inertial sensor (MetaMotionR) and a hexaphonic nylon guitar for capturing strumming gestures. To characterize this system, we compared it with a high-end commercial motion capture system (Qualisys) typically used in the controlled environments of research laboratories, in two complementary tasks: comparisons of rotational and translational data. For the rotations, we were able to compare our results with those that are found in the literature, obtaining RMSE below 10° for 88% of the curves. The translations were compared in two ways: by double derivation of positional data from the mocap and by double integration of IMU acceleration data. For the task of estimating displacements from acceleration data, we developed a compensative-integration method to deal with the oscillatory character of the strumming, whose approximative results are very dependent on the type of gestures and segmentation; a value of 0.77 was obtained for the average of the normalized covariance coefficients of the displacement magnitudes. Although not in the ideal range, these results point to a clearly acceptable trade-off between the flexibility, portability and low cost of the proposed system when compared to the limited use and cost of the high-end motion capture standard in interactive music setups.

## 1. Introduction

Musical performances with acoustic instruments and voice are often associated with electronic-digital equipment, such as an amplification system or a more complex context-sensitive signal processing in which data from different kinds of sensors are combined. The term Augmented Musical Instrument (AMI), or augmented musical practice, is used to characterize these situations. Digital Musical Instruments (DMIs), on the other hand, are fully built up from different types of sensors, at times mimicking traditional acoustic interfaces and physical vibrational sources [1]. These two broad categories are not neatly separated, for what one of the reasons is the fact that they can share a good deal of software and hardware. Augmented instruments can also be used for studying performance techniques and expressiveness in musical performances, with the Yamaha’s Disklavier as a notable example [2].

High quality sensors are normally expensive, and, in many cases, require special installations and conditions for their use. In recent years, affordable 3D Micro-Electrical-Mechanical System (MEMS), e.g., accelerometers and gyroscopes, have become available for consumer use [3]. These devices are named Inertial Measurement Unit (IMU) and can be differentiated by the number of Degrees of freedom (DoF) offered by the implemented sensors on them: 6 DoF (three-dimensional (3D) gyroscopes associated with 3D accelerometers), and 9 DoF (3D gyroscopes associated with 3D accelerometers and 3D magnetometers). The development of algorithms for the fusion of these data allows for estimating linear acceleration (isolated from gravity), angular velocities (rotation), and attitude (spatial positioning).

In the field of music and new technologies, considerable effort has been made to adequately characterize these devices, which combine flexibility with budget constraints. In the field of new interfaces for musical expression, it is not uncommon that designs are based on a good deal of intuition. In these designs, the search for personal expression and development of expertise defies standardization. On the other hand, it is also possible to find studies dedicated to a systematic review of different types of sensors for musical expression [4]. There are also hybrid initiatives that balance between these two poles, such as the present study, which associates artistic needs with the development of a research project in a short period of time.

We have conducted an experiment with a wearable 9 DoF wireless IMU, named MetaMotionR at UFMG. This experiment was extended by a comparative study using a Qualisys optical motion capture system, during a three-month research visit to McGill University. Our goal was to study the accuracy of the data generated by the sensor in a musical performance, the characteristics of its wireless transmission, and its potential for use in interactive music setups. To accomplish that, we adopted actual musical situations to compare the data that were provided by the optical motion and inertial systems to capture the performance on a nylon guitar. Several issues arose during the experiment and during data analysis which are studied in detail in this work: Bluetooth Low Energy (BLE) transmission protocol, delay in IMU response, positioning of the sensor and markers, data synchronization, and integration of acceleration curves. Thus, this study contemplates an evaluation of the effect of these issues on the IMU data, and qualitative and quantitative analysis of IMU measurements compared to the motion capture data provided by Qualisys.

Comparisons were made using rotational and translational data. The direct comparison of the attitude angles delivered by each system (rotational data) is easily addressed, in contrast to the more complex comparison of translation movements. This difference happens because selected movements include a combination of translation and rotation, which affects the linear acceleration values delivered by the inertial sensors. Thus, we propose a compensation method for the double integration of acceleration curves in cyclic movements, which may be useful in situations not requiring high accuracy. Further comparisons are performed with the derivation of positional data. This study is exploratory and presents the objective of obtaining a general qualitative view of the behavior and accuracy of the selected sensor under the conditions offered by our setup. All of the the codes of evaluation and proposed methods, as well as the datasets, are publicly available at www.musica.ufmg.br/lapis.

The paper is structured, as follows. Section 2 presents the related works that adopted motion capture systems and IMUs to analyze human movements, varying from validation to exploratory studies. In Section 3, we describe the chosen IMU, some aspects of data streaming with the BLE protocol, the Qualisys configuration used in this study, and the setup used to receive and record sensor data and audio from one guitar in one notebook. The proposed compensation method for integration is also presented here. Section 4 depicts the musical excerpts, the participants, and defines the adopted recording protocol. Section 5 describes the steps used to align the data, and also the segmentation process. The presentation of results in Section 6 includes quantitative and qualitative analysis of the data comparison. Finally, the Section 7 discusses the results and prospects for using the selected sensor in daily musical situations.

## 2. Related Work

Although the present study is exploratory and not dedicated to the validation of a specific sensor; it is precisely in this area that we can find a series of works that compare data from inertial sensors and 3D optical systems. Comparisons can be made in very controlled environments or involve participants with diverse purposes. Cuesta-Vargas et al. [5] did an extensive literature review around works that “compare inertial sensors with any kind of gold standard”, indicating that “this gold standard has to be a tool for measuring human movement”. Lee and Jung [6] proposed a new method for local frame alignment between an IMU and a motion capture system, validating it with well-controlled experiments. Ricci et al. [7] used a robotic arm and commercial IMUs to study movements related to “typical human dynamics”, and got errors of up to 10°, depending on the frequency, amplitude and orientation of the rotations.

Some validations are made based on data from sports practice [8,9], daily human activities [10], surgical practice [11], or even horse walking and trotting [12]. Several validation studies are aimed at clinical purposes and measure the angular movements of different parts of the body or gait [13,14,15,16,17,18]. In such cases, accuracy is essential to define limits between normal and impaired movements.

We encountered two works that used sensors from the same brand we have chosen for the present study. Anwary et al. [13] use IMUs made by Mbientlab for analyzing gait asymmetries, aided by an algorithm specially written for data fusion. They also use Qualisys measurements to validate the results that were obtained with the IMUs. Here, the distance estimation is made by double integration, using a method known as zero-velocity update (ZUPT). This method is justified as follows: “when a stationary period of the acceleration is detected the assumption is made that the foot is on the ground and the velocity at that time is set to 0”. The results, obtained for a young and an older group, are all above 88%.

Beange [16] conducted validation tests with MetaMotionR sensors in two different environments: a controlled one, using a motorized gimbal with rotations around the three axes, and an uncontrolled one, for the assessment of functional movement quality of the spine in patients with low back pain. Rotational data that were produced by the IMUs were compared with optical motion capture data produced by a Vicon system. The conclusions are that the IMUs “have acceptable performance in all axes when considering absolute angle orientation and motion tracking, and measurement of local dynamic stability; however, there is a low-to-moderate correlation in one non-primary axis, and that axis changes depending on the direction of motion”. This work reports the importance of proper sensor placement, and problems related to real-time streaming due to inconsistencies in the frame rate.

In all of these studies, the results depend on the experiment design (controlled environment or not, choice of sensors, type of movements), selection of participants, and intended goals. Most of them validate the angular displacement data produced by the IMUs. In clinical situations, Root-Mean-Square Errors (RMSE) of 2° or less are acceptable, and between 2 and 5° tolerable [16]. For in-field applications, Bessone et al. [8] consider that RMSE below 5° are acceptable, and below 10° tolerable.

In the music technology field, we also find studies that compare optical and inertial data, such as the ones conducted by Skogstad et al. [19] on synthesis control and by Solberg and Jensenius [20] on the analysis of dancing to electronic dance music. There are also works that are dedicated to conducting gestures, for use in interactive and multimodal environments [21], or human-robot interaction [22]. Polfreman [23] compared the recognition of hand postures, for musical purposes, using optical, inertial, and muscular information.

A few works analyze communication protocols for interactive systems to estimate the viability of them in live performances. McPherson et al. [24] studied the latencies presented by standard configurations used in these systems, while Wang et al. [25] performed a review of the capabilities and drawbacks of using BLE in musical applications. They focused on using BLE Musical Instrument Digital Interface (MIDI) when compared to other wired and wireless implementations. The BLE protocol showed higher latency values than the other option. Despite that, the authors concluded that “BLE MIDI is a potentially interesting replacement for wired MIDI interfaces due to its wireless capability and extensive hardware and software support in modern systems.”

Works dedicated to guitarists’ gestures are not very common, especially those that focus on the strumming technique. They can be grouped into: creative applications in real-time [26,27]; development of new interfaces or augmented instruments [28,29,30,31]; and, performance analysis [32,33,34,35].

The work by Visi et al. [26] is a real-time creative application, in which wristbands with flex sensors and three-axis accelerometers are used on both arms of an electric guitar player. Acceleration data produced by the strumming gesture are mapped to sound processing and “affects the timbre and decay of the strummed chord according to the intensity of the movement”. Pérez et al. [27] propose a method for extracting guitar instrumental controls in real-time by combining motion capture data, audio analysis and musical score. Guaus et al. [28] propose a gesture capture system able to gather the movements from left-hand fingers, using capacitive sensors on the fingerboard of guitars. Larsen et al. [29] developed an actuated guitar that “utilizes a normal electrical guitar, sensors to capture the rhythmic motion of alternative fully functioning limbs, such as a foot, knee, or head, and a motorized fader moving a pick back and forth across the strings”. Harrison et al. [30] developed “four guitar-derivative DMIs to be suitable for performing strummed harmonic accompaniments to a folk tune,” and studied the reactions of 32 players (half of them were competent guitarists and the other half non-musicians) to different input modalities and physical forms. Verwulgen et al. [31] implemented two types of ergonomic adaptions to the guitar design and tested them with professional players using a Vicon motion capture system.

The works focused on performance analysis usually employ sensors to characterize players or technical patterns of guitar strumming. Matsushita and Iwase [32] presents a device that is similar to a wristwatch using a three-axis gyro sensor to analyze the guitar strumming by the players. With this, they could clearly distinguish between beginners and experienced players. Freire et al. [33] describes a study of microtiming features present in accompaniment patterns played with strummed chords. Perez-Carrillo [34] presents two methods to reconstruct the trajectory of occluded markers in motion capture sessions of guitarists plucking strings: “a rigid-body model to track the motion of the guitar strings and a flexible-body model to track the motion of the hands.” Armondes et al. [35] propose a multimodal approach to the strumming technique using multichannel audio recording, an IMU with six degrees of freedom, and a high frame rate video recording to analyze the connection between gestures, rhythm, and generated sound.

## 3. Description of the Setup

This Section describes the analyzed capture systems: IMU MetaMotionR and Qualisys optical motion capture system. It also presents the details of the audio recording device GuiaRT. The proposed compensation method to integrate the acceleration data is delineated in Section 6.2.2.

### 3.1. The IMU MetaMotionR

We chose the MetaMotionR sensor for this study, because it is a light, compact, wireless, and wearable sensor manufactured by MbientLab, with a rechargeable battery. It can measure physical quantities, such as acceleration, angular velocity, magnetic field, temperature, atmospheric pressure, and ambient light [36].

#### 3.1.1. Configuration

This device uses the BLE protocol for wireless communication and offers nine degrees of freedom, provided by the three axes of the built-in accelerometer, gyroscope, and magnetometer. A fusion algorithm developed by Bosch is also provided with the unit, which allows for estimating linear acceleration and spatial attitude. It can operate at a sampling rate of 800 Hz with internal storage and up to 100 Hz with wireless streaming. The accelerometer and gyroscope are capable of transmitting data at 100 Hz, the magnetometer at 25 Hz. There is no way to determine the sample rate of the fusion algorithm during transmission, which is determined by its low-level internal routines. We were able to achieve a sampling rate of 100 Hz using one or two sensors. The firmware version used is 1.5.0.

The fusion algorithm has four modes of operation, each combining the individual sensors differently: NDoF (estimates absolute orientation with all three sensors), IMUPlus (estimates relative orientation in space using data from the accelerometer and gyroscope), Compass (combines accelerometer and magnetometer), and M4G (uses the magnetometer to detect rotation). By empirical analysis, we adopted the IMUPlus mode, due to the higher sample rate and the fact that absolute orientation is usually not a problem for live musical performances. However, even the use of the magnetometer for absolute orientation does not prevent yaw drifts, as shown by [37]. We opt to receive attitude data as Euler angles, as they are the default units for rotations in Qualisys, and they are more intuitive for performance than quaternions.

We designed a script in Swift language, adapting the Software Development Kit (SDK) code provided by the company and adding the SwiftOSC library [38], written by Devin Roth, to allow the use of the Open Sound Control (OSC) protocol, in order to use the MetaMotionR sensor in streaming mode. OSC is a protocol for networking sound devices, computers, and other multimedia resources for live performance, developed by Center for New Music and Audio Technologies (CNMAT), at the University of California, Berkeley [39].

This script prepares the sensor to send data in the chosen fusion mode and, once received, the data are routed to a local server using the OSC. OSC messages are received using the Max/MSP programming language.

#### 3.1.2. Effective Transmission Rate with BLE

The BLE protocol has its origins in the first decade of the 21st century and aims to enable products that require less current consumption, less complexity, and less cost than (Bluetooth) Basic Rate/Enhanced Data Rate (BR/EDR) [40]. BLE is based on connection events that allow devices in use to enter a lower power mode (or inactivity) when the transmission/reception of an event is complete, operating more efficiently on limited bandwidth. In this type of wireless transmission, several factors can affect its performance, e.g., the different pieces of hardware in use, software versions, distances, environmental interference, or the battery charge level.

In the present study, we used a 2019 MacBook Pro 13’, with a 2.4 GHz processor Intel Core i5, running MacOS 10.14, and Max/MSP version 8 (with signal vector size set to 64 samples, scheduler in overdrive and messages at audio interrupt). In the Max/MSP patch, the incoming data consists of ten 16-bit values for three different physical quantities: three values for linear acceleration (expressed in *g*), three for angular velocity (expressed in degrees per second), four for Euler angles (expressed in degrees, one for roll, other for pitch, and two identical values for yaw/heading). Each OSC message consists of a complete set of values for each quantity. The Max/MSP patch also has a monitor for checking the actual sample rate of the incoming data, and the number of messages received is counted and displayed as a curve, which slightly oscillates around 100 Hz. The patch timestamps the incoming data at its arrival, with a resolution of 1 ms, and records it in a buffer.

**Figure 1:**
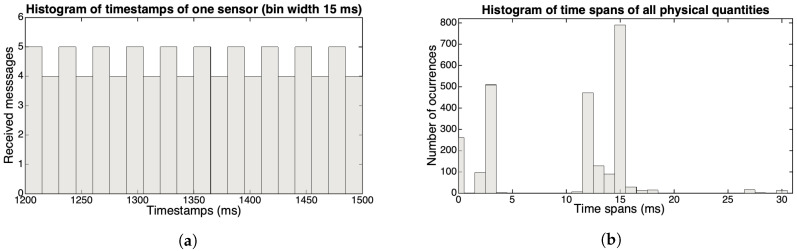
Time spans between IMU incoming messages. (**a**) Histogram of timestamps of one sensor with a bin width of 15 ms; (**b**) Histogram of time spans between subsequent messages of all physical quantities in a particular take.

**Figure 2:**
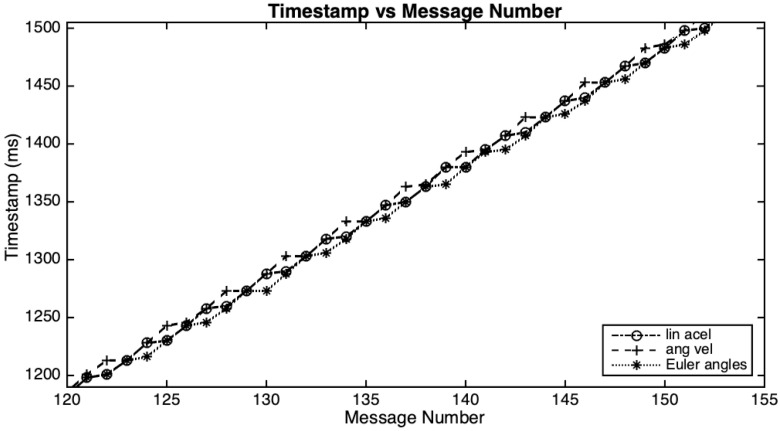
Plot of timestamp vs. message number for each physical quantity.

Looking in more detail at the time spans between two subsequent messages of each physical quantity in a particular take, it is possible to note that the most present values are 0, 3, 11, and 15 ms (see the histogram in Figure 1a) and that the overall average for time spans is 9.88 ms. These irregularities are intrinsic to the BLE protocol. If we now look at the timestamps of all messages, then we can see a new aspect of the data transfer. Arrivals (connection events) occur rather regularly every 15 ms, and new values for one or two of the quantities appear twice (which explains the zero interval time mentioned above), and continue to alternate this pattern. Figure 2 plots the timestamp of each message (vertical axis) versus its number during 300 ms of the same take. The histogram of the same timestamps, while using a bin width of 15 ms, is depicted in Figure 1b, where it is possible to observe the alternation between 4 and 5 message arrivals. Although there are variations in the timing of this reception process (as Figure 1b shows), likely most connection intervals used by this setup with a MacBook Pro is between 11.25 and 15 ms, values that are according to Kianoosh Karami [41].

#### 3.1.3. Rebuilding the Sample Rate

We could reconstruct the sample rate of 100 Hz using a timer and a filterv based on the previous observations. Every 10 ms, a new value is generated for each dimension, on each physical quantity, as the output of a moving average filter. Hence, the average time span between messages is just below 10 ms, and the maximal detected interval time was 30 ms (occurring very rarely), we have chosen a three-point filter. This filter is directly fed by the arrival data of each quantity and by the repetition of the last value when the arrival time span exceeds 10 ms (the timer rate). This action prevents the repetition of two identical values on the filter output. The synchronized data are also recorded in a separate buffer.

#### 3.1.4. An Approximate Estimation of the IMU Response Delay

Another critical issue in evaluating a sensor for live performances is its response delay, which is, the time elapsed between a given action and the reception of the sensor response by the main computer or processor. Based on the above discussion on transmission rate, a fixed value is not expected because of several layers of latency and jitter in the data capture and transmission, e.g., delays in the response of physical components, processing, conditioning, and transmission times.

**Figure 3:**
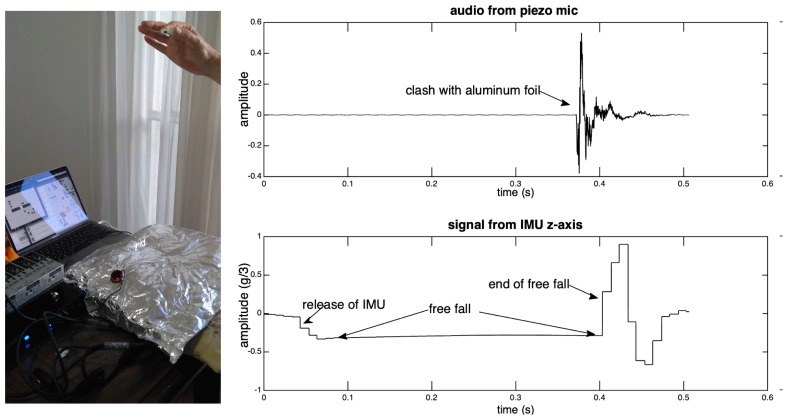
Measurement of the IMU response delay. On the left, preparation for the free fall. On the right, two-channel recording with audio and sensor data.

We designed an experiment to empirically calculate the MetaMotionR response delay due to the estimation of linear acceleration, using the equipment selected for this study. The sensor was put in free fall in the z-axis direction from a height of about 60 cm (a safe distance to allow the acceleration of gravity to be reached, without much risk of turning), and fell off on a cushion covered with aluminum foil. On this foil, there was a contact microphone plugged into the audio interface. In Max/MSP, we recorded two channels of audio, one with the sound generated by the impact and the other with the acceleration data delivered by the sensor. Subsequently, we manually extracted the time elapsed between the impact and the sensor response (see Figure 3), and average the values that are given by two hundred repetitions of this procedure.

The observed average result was 24.4±6.4 ms, and it is possible to observe a strong correlation between its standard deviation and the connection interval of our setup, of 11.25–15 ms. This procedure can offer the user a value for the sensor response estimated in conditions that are similar to real performance situations. This value will also be used in the time alignment of data, as described in Section 5.1.

#### 3.1.5. Effective Transmission Rate with Two Sensors

In the Mbientlab discussion forum, one can read that the effective sample for streaming data depends on the number of sensors connected, getting smaller with additional sensors that are connected through WiFi hub with USB dongles [42].

Although we used only one sensor in the current study, we experimented with two of them (the amount that we intend to use for live performances) to estimate the effective sample rate we could count on in such a situation. We were able to stream data at 100 Hz, with a little more variation in the time spans for each quantity. The average interval was 9.86 ms for one sensor and 9.92 ms for the other. The histogram of the timestamps of all physical quantities send by the two sensors during 500 ms of the test can be seen in Figure S1. The connection intervals seem to remain the same, but with more messages being sent at each interval.

### 3.2. Multichannel Audio Recording with GuiaRT

GuiaRT is a real-time setup built on a nylon guitar with hexaphonic pickups, developed under the supervision of the first author [43]. It is used not only as an AMI in interactive environments but also in the study of performances. In addition to the modified guitar, the setup also includes signal conditioning, audio conversion, and a program running in Max/MSP [44].

For the present study, the same program receives data from the IMU and the pickups, recording both synchronously when the pedal is triggered.

GuiaRT extracts low-level descriptors, such as start time, string number, fundamental frequency, fret number, amplitude, spectral centroid, the presence of slurs, pizzicato, harmonics, bend, vibrato and microtuning, and also some mid-level features [43,45,46].

The version of GuiaRT for this study used the notebook and the Max/MSP 8 software mentioned in Section 3.1.2, connected to a Presonus FireStudio audio interface with a sample rate of 48 kHz. Impedance matching and signal balancing were made with Behringer DI-20 direct boxes.

### 3.3. Qualisys Motion Capture System

Qualisys is an optical motion capture system that consists of a set of near-infrared cameras connected through Ethernet. Each camera emits infrared light, which is reflected by passive markers generating two-dimensional (2D) positional data. The proprietary software collects this 2D data and computes the three-dimensional (3D) positions of these markers in space.

The infrastructure used for this work includes two Oqus 300, four Oqus 400, four Oqus 700 cameras, and Qualisys recording and editing software Qualisys Track Manager (QTM) [47] version number 2019.1 (build 4420).

We placed markers on the IMU, right thumbnail (close to the contact point between the pick—or finger—and strings during the strum), and guitar since this study is focused on the guitar playing gestures. Passive markers of size 7mm were used in the IMU, while 10mm markers were attached to the thumbnail. The sampling rate set for the capture was 100 Hz, being associated with a video recording (including audio) of 25 fps.

#### Rigid Bodies

**Figure 4:**
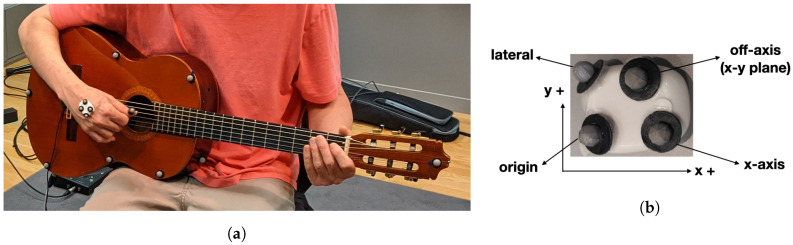
Guitar and IMU used in this study which were set as rigid bodies in the Qualisys motion capture system. (**a**) Guitar with markers; (**b**) IMU with markers.

The guitar was mapped as a rigid body with 14 markers of size 13 mm (Figure 4a). The IMU was also mapped as a rigid body, with four markers, as can be seen in Figure 4b.

The IMU x- and y-axes were aligned with the Qualisys L-shaped reference structure used for the calibration so that the four markers have the coordinates that are shown in Table 1. The definition of Euler angles used the option: “All three rotation axes are the same as the reference axes—the second and third rotation axes are not affected by the first or second rotations” ([47], p. 187), to make them compatible with the angles internally generated by the IMU. For the same reason, the IMU rigid body was translated to its origin point and reoriented in the QTM software, according to Figure 4b. The IMU origin point was considered to be the origin of its frame of reference, around which the rotations are calculated.

### 3.4. Proposed Compensation Method for Integrating the Acceleration Data

We designed tools for the integration of the acceleration data in order to estimate the linear displacement in each axis. These tools are focused on the gestures under examination, which is, periodic (or quasi) strums on the guitar. We start with a simple example, which presents a procedure to balance acceleration cycles, and we then proceed with a more complex one, which also includes rotation. Strategies, kike ZUPT, used in gait analyses [13], do not apply in movements, such as strumming on a guitar, since it is not possible to associate periods of zero acceleration with the absence of speed. The strumming tecnique has an oscillatory character, which means that it is related to the simple harmonic motion, in which position, velocity, and acceleration follow the same pattern, but with different phases.

**Figure 5:**
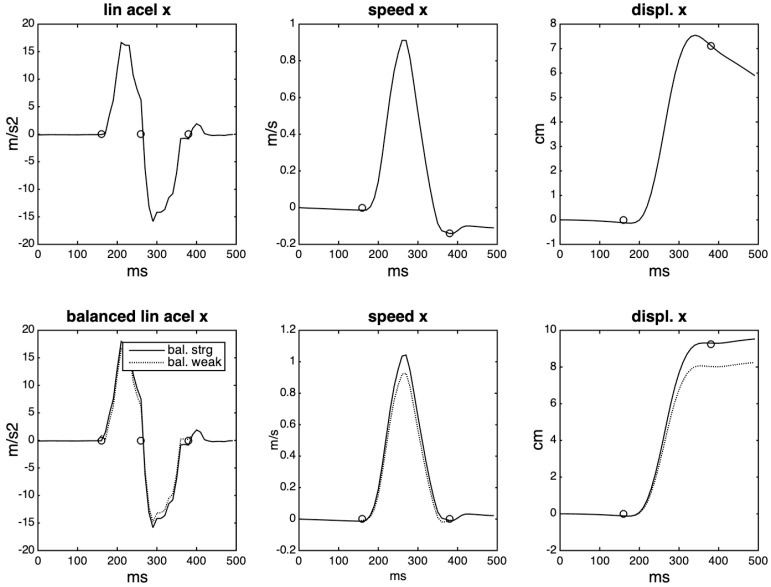
Double integration of the acceleration curve of a simple linear trajectory. On the second row, the solid line indicates a balance towards the strong lobe, the dashed line a balance towards the weaker lobe. The small circles indicate the zero-crossings extracted from the acceleration curve.

A simple linear trajectory has a one-lobe curve for the speed—which starts on zero, goes up or down to maximal speed, and returns to zero—and a symmetric (or almost) two-lobe curve for the acceleration: an acceleration followed by a deceleration in the chosen direction, with the maximal speed at the zero-crossing. Sliding the IMU in the positive direction of its x-axis produces a curve like the first one depicted in Figure 5. The values are multiplied by the gravity constant of 9.81. The integration of this curve using the trapezoidal method gives an estimation of the speed, and a second integration gives the displacement. The first row of the figure illustrates this process and reveals a common problem with this procedure: it is challenging for humans to generate a fully symmetric acceleration curve, because the applied force, due to diverse factors, usually is not symmetrical. Additionally, the lobes in each cycle can be represented by a different number of samples. Therefore, we have chosen to apply a compensating factor to the weaker (or stronger) lobe, based on the number of points and mean values of each lobe. Both options are shown in the figure. Before this compensation, it is necessary to define the boundaries of the curve through the detection of three zero-crossings: at the beginning, middle, and end of the gesture. The results of applying this procedure can be seen in the second row of the same figure. The balancing procedure allows us to zero the speed at the end of the acceleration cycle.

#### 3.4.1. Displacement with Rotations

Most hand gestures combine translation and rotation in many different ways, including changes of shape, coordination with other parts and control of dynamic forces, subjects that are not covered by the authors’ expertise, and also outside the scope of this text. We performed the analysis of data delivered by the IMU during an oscillatory motion (related to the strum gesture), before delving into the dynamic data captured during a musical situation.

**Figure 6:**
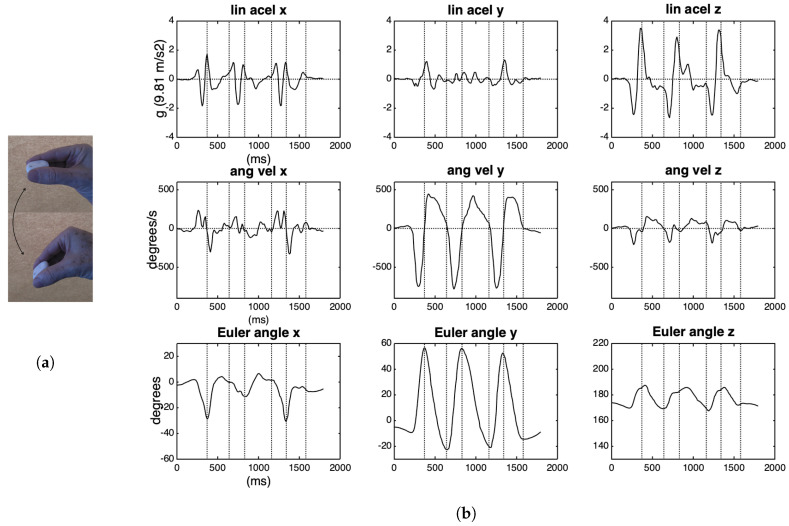
IMU data from six wrist rotations. (**a**) Wrist rotation. (**b**) Curves of three-dimensional (3D) accelerations, angular velocities and Euler angles of wrist rotations.

We recorded in Max/MSP a series of six wrist rotations (three down and three up) with the IMU held between the thumb and forefinger, its xy plane parallel to the floor, and z-axis pointing up, as depicted in Figure 6a. In a very schematic way, this gesture consists of a rotation around the y-axis, and a translation taking place mainly in the z-axis.

Figure 6b shows the curves in the three axes for linear acceleration, angular velocity, and attitude (Euler angles). It can be noted that the curves with the highest amplitudes are the ones just mentioned (y-rotation and z-acceleration). The dotted vertical lines are aligned with the change of direction of the rotation of the y-axis. Although these six gestures were intended to be regularly performed, the data presents a temporal asymmetry, with the downward gestures being faster than the upward ones. Another point that draws attention immediately is the reverse signal between angular velocity and attitude, which probably comes from the IMU firmware. In each axis, a positive angular displacement corresponds to a negative speed, and vice-versa.

In cyclical movements, there is a partial overlap of the acceleration curves of the subsequent gestures, the deceleration of the former already contributing to the acceleration of the next gesture. Therefore, only the first and the last gestures in a sequence would have a lobe of acceleration not shared with the others. This overlap is quite evident in faster and more regular periodic gestures, but not so evident in slower, or more irregular oscillations. For example, in Figure 6, it is possible to see small positive and negative peaks between two zero-crossings in the z-axis acceleration curve during the rotation occurring around 1000 ms. This observation helps to decide which balancing method to use: if there is a substantial overlap, then we can balance to the stronger lobe; otherwise, the balance must be based on the weaker lobe, in order to avoid a virtual and artificial injection of energy.

**Figure 7:**
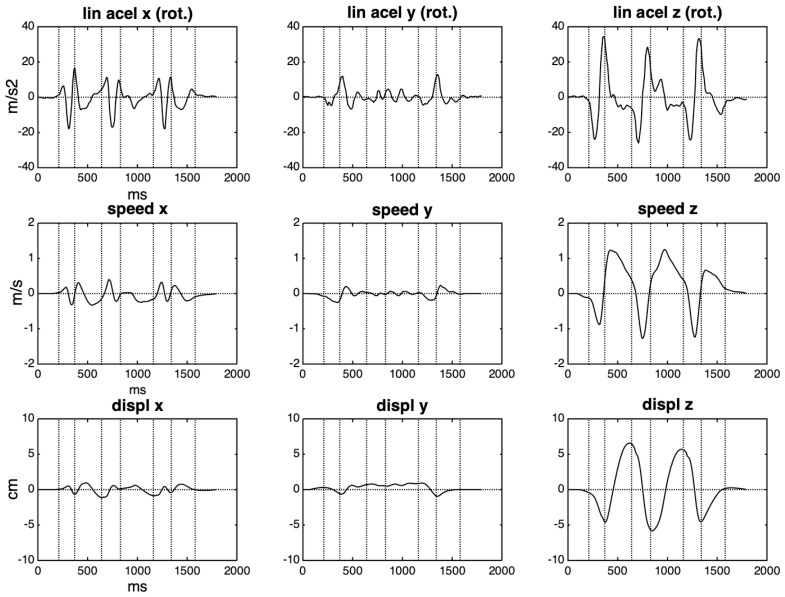
In the first row, curves of 3D acceleration rotated to the IMU frame of reference. The following rows depict the speed and displacement curves that were obtained by integration.

The acceleration on the x-axis shows a small positive rise and no compensation on the negative side. If related to a linear movement, this would indicate a movement without rest, which is not the case. A more reliable picture of what happens with linear acceleration is to rotate its values according to a fixed frame of reference. Here, the IMU itself provides this fixed frame in its resting position at the beginning of the movement; the rotation matrices are calculated from the Euler angles for each time point. The result of this rotation can be seen in the first row of Figure 7. After that, positive and negative lobes in the acceleration can be detected in each axis, although they are still considerably asymmetric.

After detecting zero-crossings in the acceleration curve of each axis (actually, crossings of a ± threshold range, with a minimum floor value) we divided the curve into two groups of partially overlapping cycles, one with its first lobe with a negative sign and the another with the first lobe with a positive sign. Then, we applied the same compensation procedure for each cycle, as described above, choosing to balance according to the weaker lobe. The estimation of the speed is done by averaging these two groups of curves, and it can be seen in the second row of Figure 7, and in more detail in Figure S2. The same procedure was applied to estimate the displacement in each axis, depicted in the third row.

We observed that small variations in the starting points already lead to very different integration curves, impairing the direct comparison with the 3D data provided by the motion capture system. In such cases, we will make the comparison in another way, by the double derivation of positional data.

These procedures for rotating the acceleration values, finding zero intersections, balancing cycles, integrating/deriving time series will be the basis for the following analyzes.

## 4. Participants, Excerpts, and Recording Procedure

Given the main purpose of this short-term study, we have chosen to collect data in a simulation of real musical situations, focused on gestures that were related to percussion, with not very rigid control over the performances. The choice of a classical guitar with hexaphonic pickups has a few reasons: the development of augmented nylon guitars in the two partner institutions (McGill and UFMG), the fact that some researchers are also guitarists, and the possibility of further analysis including the multichannel audio recordings. The musical excerpts were remotely defined by one of the authors, and two others were responsible for the performances. Such a procedure is not uncommon in our laboratories, focused on the development and use of new musical interfaces. After recording several versions of each excerpt, each musician chose the two best renditions of each passage. For the objectives of the present study, which do not include performance analyses or gesture characterization, the data provided by two semi-professional players is sufficient. Musician 1 is left-handed, and Musician 2 is right-handed; both played on the same instrument.

### 4.1. Excerpts and Takes

**Figure 8:**
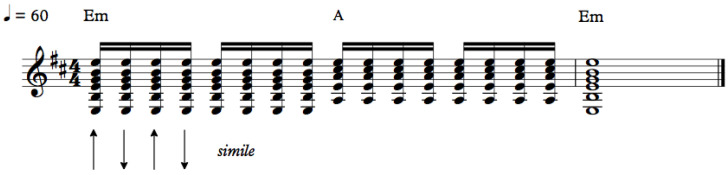
Musical excerpt 1.

**Figure 9:**
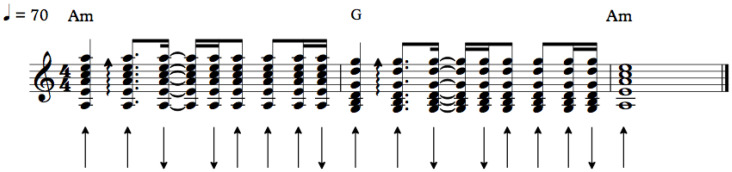
Musical excerpt 2.

**Figure 10:**
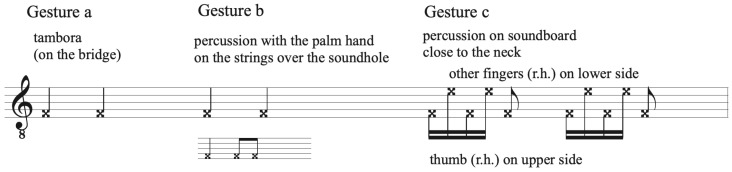
Musical excerpt 3.

The chosen musical excerpts are: (1) a sequence of down-and-up strummed chords, with a regular rhythm, based on Pink Floyd’s *Breathe* (Figure 8), with a duration of four seconds; (2) a more complex rhythmic pattern, typical from rock accompaniment, to be interpreted with some freedom (Figure 9), with a duration of ca. eight seconds; and, (3) a sequence of three different percussive musical phrases on the guitar (Figure 10), with durations between one and three seconds.

The first and second excerpts explore cyclic and relatively fast down-and-up movements of the right hand. The gestures in the first excerpt are to be played with the frequency of 120 cycles per minute, and some gestures in the second excerpt are done with a frequency of 140 cycles per minute.

The takes will be referred with a combination of letters and numbers, relative to the musician (m1 or m2), excerpt (r1, r2, or r3), and take (t1 or t2). For example, m1r1t2 indicates the second take of the excerpt (rhythm) one made by the first musician. Excerpts 1 and 2 were played by Musician 1 with a pick and by Musician 2 without a pick.

### 4.2. Recording Procedure

**Figure 11:**
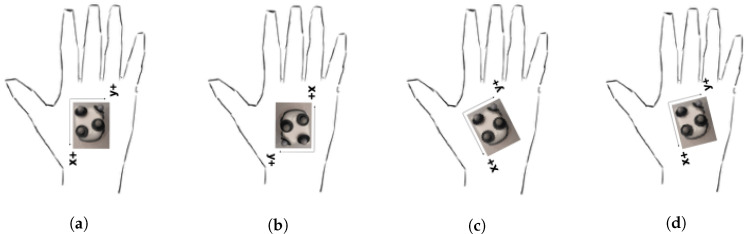
Different IMU positions used by the musicians to perform the musical excerpts. (**a**) Used by Musician 2 in excerpts 1 and 2; (**b**) Used by Musician 1 in excerpts 1 and 2; (**c**) Used by Musician 1 in excerpt 3; (**d**) Used by Musician 2 in excerpt 3.

Recordings in the systems Qualisys and GuiaRT/IMU were made independently. The musician’s chair was positioned on the origin of the reference framework, facing the positive x-axis. When the motion capture cameras started recording, the musician pressed a pedal, which started recording in Max/MSP, along with a click played on the loudspeakers. After four clicks, the performance started. The capture duration was predefined in the motion capture software QTM; for the guitar and IMU, the release of the pedal marked the end. The IMU was positioned in different ways, with double-sided adhesive tape, to test whether different orientations around the z-axis could influence the results, as depicted in Figure 11. We observed that some positions of the right hand of guitarists favor the occurrence of confusion between the axes (gimbal lock). Thus, this factor was taken into consideration during the sensor positioning to avoid creating singularities in the calculation of the rotation angles.

The data that were recorded on both systems were transferred to Matlab, and each take was stored as a data structure. All analyzes were performed on this platform after a processing phase.

## 5. Data Processing

This section outlines the process of the data yielded by the IMU and Qualisys capture systems. This process consists of a data alignment phase, and an excerpt segmentation to allow a more accurate analysis. Specific settings for filters and thresholds are also presented in this section.

### 5.1. Aligning the Data

The first step to align the data from both systems is to estimate the offset between the audio recordings, the delay between the audio recorded on video by Qualisys, and the audio recorded on GuiaRT. After that, we also estimated the distance between the guitar and the camera at 4.5 m. This distance corresponds to a delay of approximately 13 ms to be compensated from the offset value. The IMU response delay was fixed as 24.4 ms, according to the empirical analysis in Section 3.1.4). Finally, we empirically observed that the best alignments were achieved with one video frame delay, which is, 40 ms.

Therefore, the offset between the audio recordings must be compensated by these three values, in order to achieve the time alignment between the data from the motion capture system and the IMU. The result of this operation is rounded to the nearest multiple of 10 ms (as the sample rate is 100 Hz) and then applied to shift back the motion capture data.

The attitude angles also needed some alignment. The first step was to invert the signal of the IMU angles for the reasons presented above (Section 3.4.1). In addition, as we were not dealing with the absolute orientation of the rigid bodies, and also using different positions of the IMU on the hand, it was also necessary to align the Euler angles with the z-axis for each take. This procedure was done by observing the minimal and maximal values of this physical quantity, from which an offset was estimated, and further refined by visual inspection of the curves. All of the extrinsic rotations were done in the *xyz* order.

For the conversion of the acceleration values delivered by the IMU in *g* (acceleration of gravity), we used the value 9.81 m${}^{2}$/s. Values are expressed in degrees for angles, in m/s for speeds, and in cm for displacements. The IMU origin point (Figure 4b) was considered to be the origin of its frame of reference, around which the rotations are calculated.

The occlusion is an intrinsic problem of optical capture systems, which was handled in this study by performing a linear interpolation between the nearest captured points.

### 5.2. Segmentation

In performances, musicians widely explore two types of control: attacks (sudden bursts of energy) and continuous modulations of some physical or acoustical characteristics. A sensor is expected to provide both types of information when connected to specific parts of the body. In the present case, the attacks are expressed by sudden variations in acceleration and angular velocity, while the modulations are linked to the attitude angles. In situations where the sensor fusion mode is not used, gravity is used instead.

However, accelerometers are not suitable for detecting slow translations (without rotations), as in these cases, the acceleration values would be blurred with the background noise. This problem is the reason why we decided to divide the third excerpt into three different parts before the analysis, as it demands a gradual translation of the right hand from the bridge to a region close to the guitar neck.

Excerpts 1 and 2 will be analyzed as a whole from the 100 ms before the first attack to the moment of the last attack; the duration of them are 4000 ms and 8000 ms, respectively. In excerpt 3, rotation comparison will be made for each segment; for translations, this will be done for each single (as in gestures *a* and *b*) or compound attack (as in gesture *c*).

## 6. Results

The obtained results are presented in this section, as well as highlights regarding insights accomplished in each evaluation. A discussion about the overall approach is presented in Section 7. We will divide them into two topics, depending on the IMU’s physical quantity under analysis: rotational data (from attitude angles), and translation data (from linear accelerations).

The comparison of different time series was based on RMSE and normalized cross-covariances, both calculated with lags. We decided to use the two methods, because low RMSE values may also appear in weakly correlated series, and vice versa. We have found reference values in the literature only for rotational data, as comparisons based on double integration or derivation are less common. We also observed the differences between maximal and minimal values present in each time series, as indicated by Δmax, which are useful to interpret some results. The choice for covariance, and not for correlation, is justified by its greater sensitivity to differences in scale. In addition, normalized values may be useful for comparisons between curves generated by different physical quantities, as accelerations and displacements in the translations. The maximum value for the lags used in all comparisons was 12. This value corresponds to an average delay of 120 ms between the time series, a value far beyond acceptance for live performances. The tables with all comparison results can be found in Tables S1–S12. In the following subsections, we will present the results from selected takes from musicians 1 and 2.

### 6.1. Comparison of Rotational Data

The comparison between the rotational data from both systems is performed after the time alignment (see Section 5.1), and a yaw shift performed for each take. It is important to remember that the angles to be compared are expressed in relation to a moving reference frame, that is, the IMU itself. Table 2 depicts selected results obtained for rotations in each axis, and the mean value and standard deviation for each time series. The complete comparison shows 73% of RMSE below 5°, and 88% below 10°, as well as two-thirds of the covariance values above 0.95. The highest RMSE and lowest covariance values are found in the z-axis rotations. This axis also shows higher values for lags. As the sensor was positioned for each musician with a 180° rotation (see Figure 11), the z-axis mean values have similar values with opposite signs. The standard deviations can be associated with different techniques used for the strumming patterns (excerpts 1 and 2). Musician 1 has higher values for the x-axis, indicating rotational movements mainly occurring in the sagittal plane. On the other hand, Musician 2 presents higher values not only for this axis, but also for the z-axis, indicating heavier rotations also related to the vertical plane.

**Figure 12:**
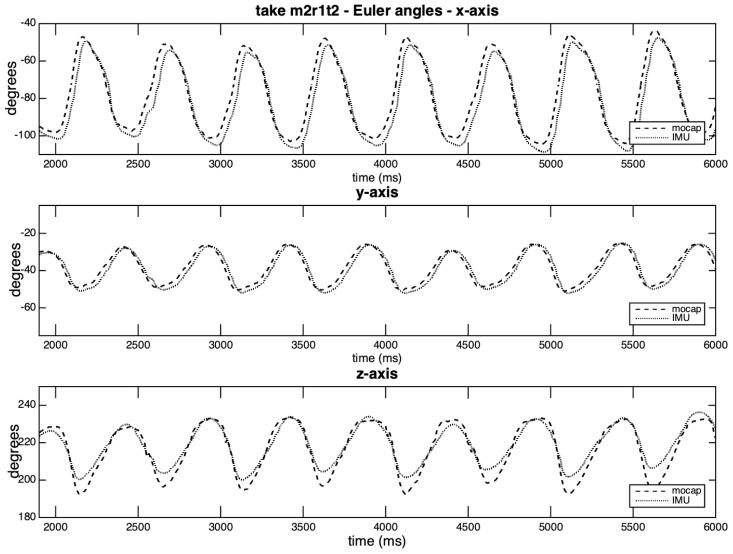
Rotation curves per axis in take m2r1t2.

**Figure 13:**
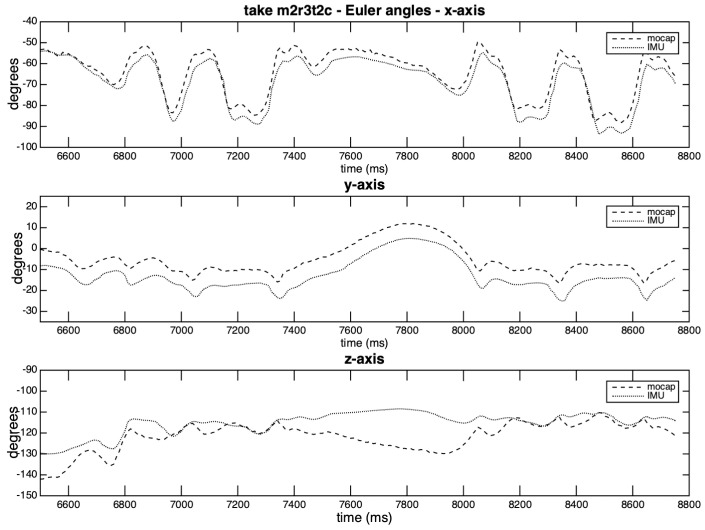
Rotation curves per axis in take m2r3t2c.

Figure 12 and Figure 13 depict the curves generated by the take exhibiting the highest covariation values for the three axes (m2r1t2), and by the take with the lowest individual covariation value (z-axis in m2r3t2c). In the Figure 12, we observe the lag of two points in axes *x* and *y*, and slight differences in amplitudes in the z-axis. There is a high correlation between the curves, which may be explained by the planar movement characteristics and a possible locking between the axes.

Figure 13 shows the substantial similarity between the curves on axes *x* and *y*, around which this particular percussive hand movement occurs. The offsets in x- and y- axes affect the RMSE without substantially affecting the covariance results, and are probably generated by the positioning of the IMU. The position of the right hand during the strumming favors some confusion between the axes. In the z-axis, there is only coincidence of peaks; although its RMSE is close to that of y-axis, their covariation coefficients are very different. This example demonstrates that drifts in attitude angles due to the soft translation of the IMU are present, but not limited to the z-axis.

### 6.2. Comparison of Translations

There is no direct way of comparing the 3D positional data that are generated by Qualisys with the 3D linear accelerations delivered by the IMU. Therefore, we will transform them in the same unit using two different methods: one from the positional data that leads to acceleration (by double derivation), and another from the acceleration data to displacement (using the proposed compensative-integration method).

#### 6.2.1. Deriving the Positional Data

To delineate a comparative analysis of the obtained results using the proposed method, we twice derived the positional data of the marker fixed on the IMU origin (see Figure 4b). We used a 6-point moving average filter for the first step, and of a five-point one for the second step. The IMU acceleration data was rotated in relation to a fixed reference frame (using the rotation matrices calculated by QTM) and passed through an eight-point moving average filter. Table 3 and Table 4 show the results of the statistical comparison between the resulting time series of each axis.

In excerpts 1 and 2, both of the musicians achieved high covariance values for axes *y* and *z*. The values obtained for the x-axis show a weaker correlation, especially for Musician 2. This axis is less explored in the strumming technique, which is primarily a 2D movement occurring in the vertical plane. More precise values for the IMU acceleration on these axes may be impaired by the fact that they depend on the z-axis rotation, which showed the worst correlation in the comparison of rotations (see Section 6.1). Another factor that may affect these results is the fact we fixed the position of the chair for the performance, but not the spatial orientation of the guitar, which is something highly personal.

Similar reasons may explain the lack of correlation in some axes in the third excerpt, with the addition that gesture *c* is more related to rotation than linear acceleration (see Figure 10). We can observe significant differences between the maximal amplitudes in each axis for each musician, which is probably linked to different performance strategies.

**Figure 14:**
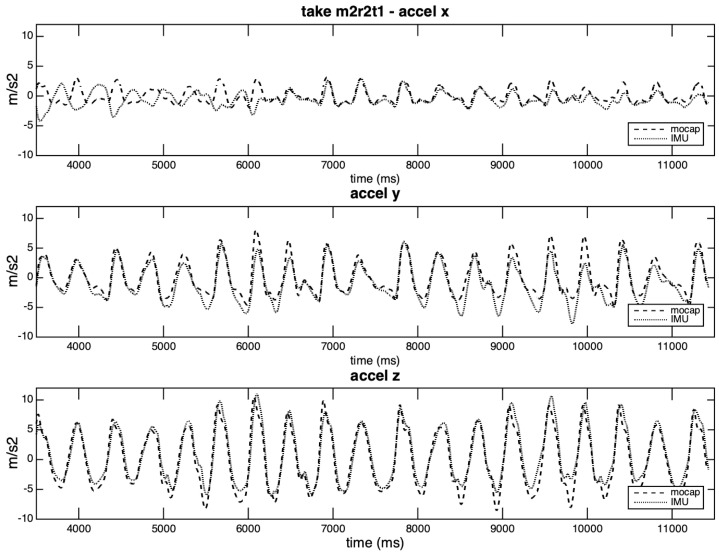
Acceleration curves per axis in take m2r2t1.

Although on a smaller scale, the small size of the rigid body (when compared with the entire volume captured by Qualisys) may also affect the accuracy of the rotation matrices. This fact can be illustrated by the first take of excerpt two played by Musician 2, which shows very good correlation values for axes *y* and *z*, and a lower value for axis *x* (the one with the lowest amplitudes). Figure 14 shows the three curves for each system. The IMU x-axis curve starts out of phase with the Qualisys curve, then enters in phase and, after a while, loses the phase again.

#### 6.2.2. Integrating the Acceleration Data

The proposed compensation method to perform the double integration of acceleration data was only applied to excerpts 1 and 2, for the reasons that are related to the difficulties in the segmentation process posed by soft translations (described in Section 3.4.1 and Section 5.2). We made an analysis for each axis and the magnitude vector (resultant vector) of the 3D displacements.

After rotating the IMU acceleration curves with the rotation matrices from Qualisys, we applied a 10-point moving average filter. The same filter was also applied to the 3D Qualisys positional data. These filters are useful to reduce the noise that is present in the acceleration measurements, which can lead to considerable drift after the integration process. These windowing filters are simpler, popular, and easier to adopt when compared with other FIR methods, as well as provide an effective way to smooth out noisy time series. After filtering, we integrated the IMU curves with the method described in Section 3.4.1, using both options for balancing the lobes. The zero-crossings were estimated using a threshold of 15% of the maximal peak in each time series, which is very effective for the curves of the most explored axes. The floor values are 0.2 for acceleration (m/s${}^{2}$), and 0.03 for speed (m/s), and help to avoid small fluctuations around zero on the less explored axes that were not removed by the filtering process. These values were empirically defined based on the observation of the zero-crossing results. The results are depicted in Table 5 and Table 6. We can observe a fair approximation (covariance above 0.8) for axes *y* and *z* in excerpt 1. Here, the small RMSE in the x-axis could mislead the interpretation. In this case, the observations about the quite planar characteristic of the strumming gesture also apply, and the differences on the x-axis between both musicians may relate to technical idiosyncrasies.

**Figure 15:**
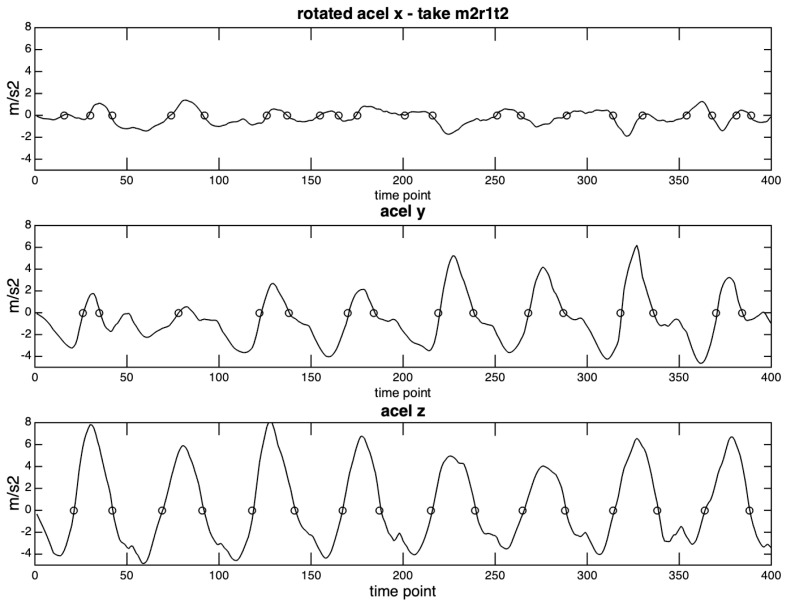
3D acceleration curves and zero-crossings from take m2r1t2.

Figure 15 shows the rotated acceleration curves for take m2r1t2, and the contrasting patterns of zero-crossings caused by differences in amplitude and regularity in each axis.

In excerpt 2, Musician 1 presents covariance values that are considerably smaller than Musician 2. This disparity can be explained by the different strategies used in the performance: Musician 1 associated the gestures with the rhythm (which has varied intervals between attacks), while Musician 2 kept oscillating the hand in short regular intervals and attacked when necessary. In the latter case, more regular acceleration cycles were available for a more precise integration process of the entire excerpt.

**Figure 16:**
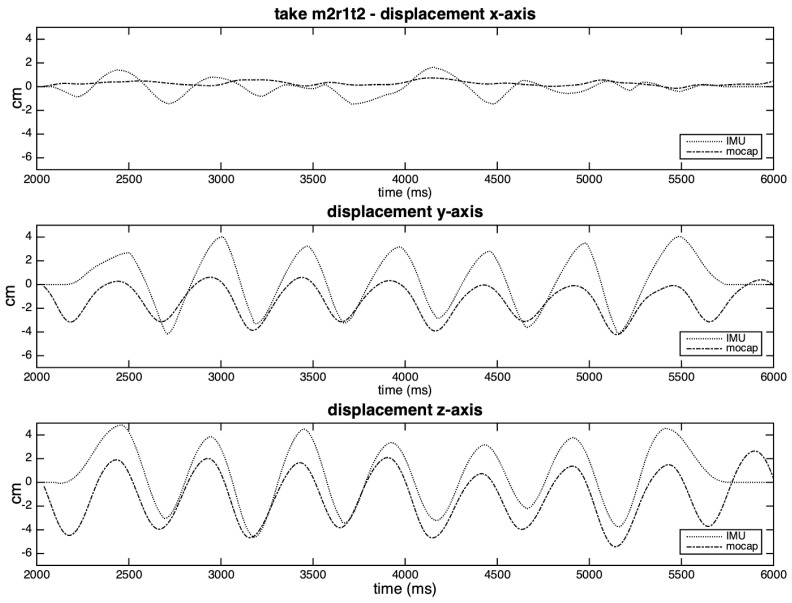
Displacement curves estimated for each axis of take m2r1t2, balanced according to the stronger lobe.

Figure 16 shows the results of the integration process to take m2r1t2, which was balanced according to the stronger lobes. It is possible to observe an overestimation of the values for all axes in the IMU curves, possibly related to the inverse amplitude levels. Not only the inaccuracies in detecting zero-crossings (see Figure 15), but also the flattening of the IMU curves at the beginning and end of the excerpt influences the overall results.

**Figure 17:**
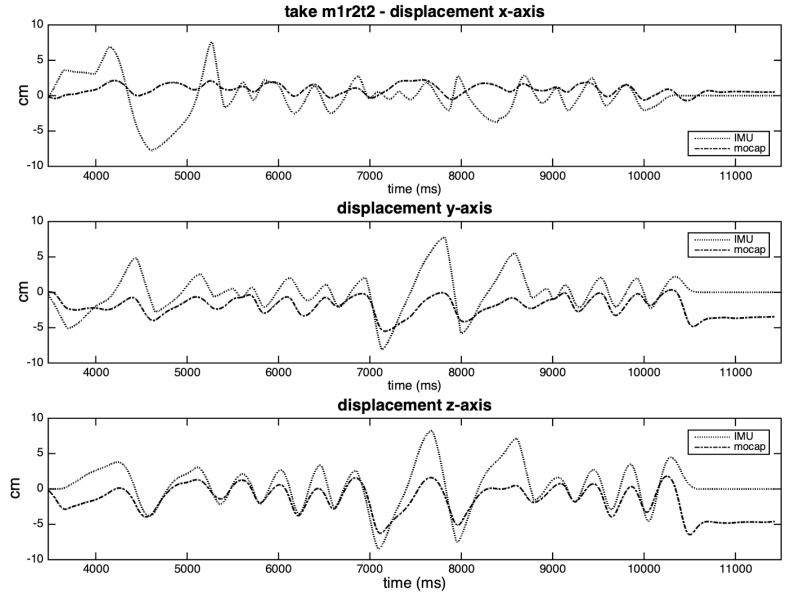
Displacement curves estimated for each axis of take **m1r2t2**, balanced according to the stronger lobe.

However, in Figure 17, we can see considerable distortions that result from the integration method to non-periodic movements. We observe that this integrating method is suitable to the moments of high periodicity of the gestures, but not accurate to the moments of relative immobility of the hand (demonstrated by wide peaks in the curves of the IMU in the figure). The complementary method (balancing with the weaker lobes) generated flatter curves instead of peaks at these same moments, as depicted in Figure S3. In these cases, the ideal would be to integrate the excerpt by shorter segments.

We used two different rotation matrices to compare the displacement magnitude (lengths of 3D positional vectors): one produced by Qualisys, the other produced by the proper IMU. In order to create a common comparison reference, the displacement values in each axis of the IMU were shifted to a minimum value of zero, before calculating their magnitude, and the values of the displacement magnitude measured by Qualisys also had their minimum values shifted to zero.

**Figure 18:**
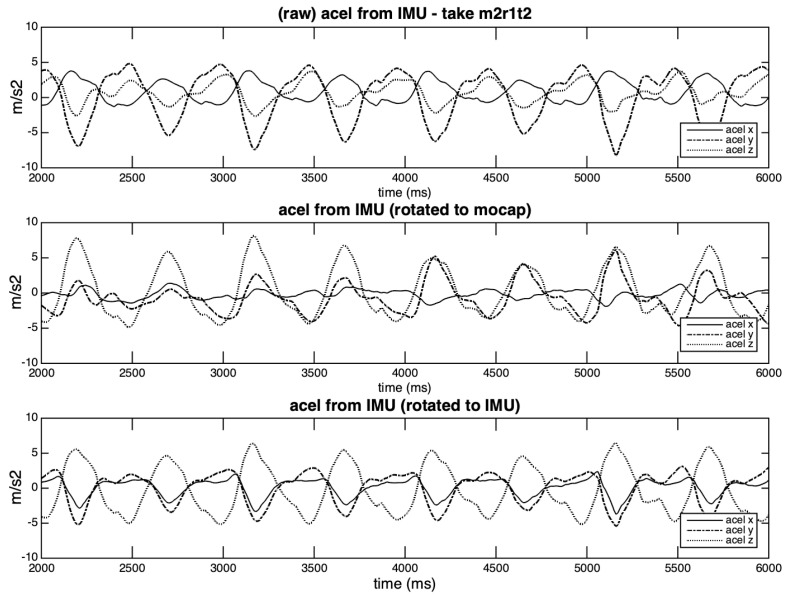
Accelerations curves from take m2r1t2: original, rotated to Qualisys angles, IMU-rotated.

Before presenting the results for this last comparison, it is important to discuss the effects of the different rotations on accelerations. The differences between the original and rotated curves from take m2r1t2 can be seen in Figure 18. The original curves (correlated, but with distinct contours) depicted in the first row are transformed in two groups with opposite phases in the third row (auto-rotated), while in the middle row (Qualisys-rotated) it is possible to observe the smaller amplitude on the x-axis, and a gradual locking of axes *y* and *z*. This inconsistency may be related to the mentioned influences of the small size of the IMU rigid body on the Qualisys rotation angles. These rotations may not affect the magnitude of the vectors represented by the 3D values. However, differences in the rotation angles and shape of the acceleration curves may alter the zero-crossing estimations and, therefore, the results.

The integration results obtained from the IMU magnitude vector using two rotation matrices and two integration methods—balancing according to the weaker lobe (w. lobe) and to the stronger lobe (s. lobe)—are summarized in Table 7. The rotations based on Qualisys angles (mocap-rotated) present higher covariance values than the ones based on the IMU angles (IMU-rotated). The differences in yaw may explain these differences: for the Qualisys reference the displacement on x-axis is minimal (see accel from IMU (rotated to mocap) in Figure 18), while with the IMU-reference *x* and *y* curves (accel from IMU (rotated to IMU) in Figure 18) are more similar (and asymmetric) and, therefore, more susceptible of integration errors. In general, the covariances that are calculated for the magnitude vectors are similar to the best results obtained by individual axes (cf. Table 5 and Table 7). The reasons for this may lie in the coplanar characteristics of the studied gestures and the similar curve patterns presented by some axes.

## 7. Discussion

We believe that the main goals of this study have been fulfilled. First, we were able to gain a deeper understanding of the BLE transmission protocol and estimate the sensor’s response delay and implement real-time streaming with a fixed sample rate. It was also possible to evaluate the data that were produced by the selected sensor in real musical situations, exploring different types of complex hand gestures. The time alignment procedure proved to be adequate for the proposed comparisons. All of the measurements are affected by the use of the MetaMotionR sensor in real-time streaming (delay, jitter, filtering), the variety of gestures, sensor positioning and, possibly, also by the size of the sensor. The markers to define it as a rigid body were placed at almost critical distances.

The three different comparisons made here—between rotations, accelerations, and displacements—have different and somewhat cumulative sources of errors. The most direct comparison, which occurred between attitude angles, is affected by yaw offsets and drifts and the axes mostly explored by the gestures. Nevertheless, we obtained results between acceptable and tolerable, when compared to the ones described in the literature [8,16].

The comparison between the accelerations, obtained by deriving positional motion capture data, is also affected by the accuracy of the rotational data, due to the use of rotation matrices, and also possibly by the guitar spatial orientation. For this type of comparison, we did not find any reference values in the literature. As they involve fewer sources of errors, these results could be used as relative references regarding the comparison of displacements, since they refer to the same translation movements.

The comparison of displacements by the integration of acceleration data is the most complex, as it depends on several factors: accuracy of the rotation angles, the effectiveness of detecting zero-crossings, type of gesture under analysis, limitations of the integration method. In this case, the results have low accuracy, which could be improved by segmentation. Furthermore, even a rough estimation of displacement during a short gesture can be very useful for interactive contexts. In the studied gestures, it was not possible to apply the assumption that was taken by ZUPT (existence of points with zero velocity and acceleration), due to the oscillatory characteristics of the strumming technique. This study’s threshold values should not be taken as reference, but as starting points, and may vary according to different musicians and gestures. For musical interaction purposes, one of the most important issues in parameterization is to offer quick and straightforward ways to test, verify, store and retrieve different configurations or presets.

In general, the data generated by the IMU showed a good correlation with the data from Qualisys, given the differences between them: kinematics versus dynamics, price, and complex versus wearable configuration. Based on this, we believe that this sensor can offer a refined control of interactive musical performances. A strong positive point for the performances is its wearable quality: wireless, small size and weight, easy to attach to different parts of the body. A significant drawback is related to the jitter in the BLE transmission, and its restricted bandwidth, which limits the number of sensors to be used. Another critical issue is the response delay, which can impair its use in DMIs: an average delay of 24 ms would not be acceptable in situations that require clear attacks.

In conclusion, we consider as main contributions of this study the implementation of a BLE streaming with a fixed sample rate, an estimation of the sensor response delay, an overview of the accuracy of the data produced by hand gestures in musical situations, and the development or adaptation of tools that can be used in interactive setups, mainly with augmented musical instruments.

## Figures and Tables

**Table 1:** Coordinates of the IMU rigid body markers to align with the Qualisys L-shaped reference.

Marker	x (mm)	y (mm)	z (mm)
origin	0	0	0
x-axis	22	0	0
off-axis	14.95	16.76	0
lateral	−2.9	20.57	−3.28

**Table 2:** RMSE and covariances (per axis) between rotational data generated by each system—selected takes from musicians 1 and 2.

Take	Axis	RMSE (°)	Lag	X-Covar	Mocap Mean (°)	Stdev	Imu Mean (°)	Stdev
m1r1t1	x	4.42	0	0.997	80.7	17.4	76.5	17.4
	y	0.68	0	0.993	29.1	5.8	29.1	5.7
	z	3.48	−8	0.877	−293.8	6.6	−293.7	5.5
m2r1t2	x	4.22	2	0.996	−78.1	19.7	−82.2	19.7
	y	1.14	2	0.996	−38.4	8.5	−39.1	8.9
	z	4.37	0	0.974	217.0	13.4	218.7	10.5
m1r2t1	x	3.24	0	0.993	83.3	17.3	80.9	17.9
	y	1.33	0	0.991	38.6	10.1	38.5	10.1
	z	6.25	−4	0.820	74.4	10.1	73.4	10.0
m2r2t2	x	6.69	−1	0.996	−79.7	14.7	−86.2	14.2
	y	0.79	−1	0.996	−42.3	9.3	−42.4	9.6
	z	5.13	−3	0.890	212.8	10.8	212.3	11.0
m1r3t1a	x	4.43	−1	0.972	−68.3	3.9	−72.5	4.4
	y	2.36	0/−1	0.994	−23.9	7.7	−26.0	7.7
	z	14.47	−1	0.839	−119.7	9.6	−106.9	3.7
m2r3t2a	x	5.68	0/1	0.948	−67.8	5.8	−73.1	6.1
	y	2.54	1	0.958	−30.6	7.9	−31.4	8.5
	z	11.20	2/1	0.730	−150.1	7.6	−140.2	6.6
m1r3t1b	x	7.87	0/−1	0.989	−73.6	9.4	−81.2	8.2
	y	4.64	−1	0.866	−4.0	1.6	0.6	0.9
	z	2.74	−1	0.987	−96.8	9.6	−94.8	9.8
m2r3t2b	x	3.29	0	0.985	−61.4	7.7	−63.6	9.6
	y	7.60	0	0.994	−8.7	9.6	−16.3	9.8
	z	13.39	2/1	0.928	−131.8	11.5	−121.6	3.1
m1r3t1c	x	11.09	−1	0.990	−76.8	8.0	−87.8	7.7
	y	1.56	−1	0.990	−1.1	4.1	−2.5	4.0
	z	6.83	−2	0.505	−93.5	4.7	−88.0	2.7
m2r3t2c	x	4.40	1	0.987	−64.4	11.0	−68.4	11.4
	y	7.64	1	0.993	−5.3	7.1	−12.9	7.4
	z	8.62	0	0.580	−121.6	6.9	−115.2	5.4

**Table 3:** Covariances (per axis) between linear accelerations from IMU and double derivatives of mocap positional data—selected takes from musicians 1 and 2.

Axis	x		y		z	
Take	x-Covar	Lag	x-Covar	Lag	x-Covar	Lag
m1r1t1	0.808	1	0.948	0	0.978	−1
m2r1t2	0.549	−3	0.876	−2	0.970	−2
m1r2t1	0.854	0	0.948	−1	0.968	−1
m2r2t2	0.447	0	0.956	1	0.989	1
m1r3t1a	0.952	0	0.899	1	0.973	0
m2r3t2a	0.787	−2	0.930	−2	0.955	−2
m1r3t1b	0.809	0	0.970	0	0.936	1
m2r3t2b	0.679	−1	0.658	−1	0.848	−1
m1r3t1c	0.719	−1	0.788	0	0.627	1
m2r3t2c	0.753	−1	0.738	−1	0.837	−1

**Table 4:** RMSE and maximal ranges (per axis) in the comparison of acceleration data of both systems—selected takes from musicians 1 and 2.

Axis	x			y			z		
	RMSE	ΔMax		RMSE	ΔMax		RMSE	ΔMax	
Take	(m/s${}^{2}$)	Mocap	Imu	(m/s${}^{2}$)	Mocap	Imu	(m/s${}^{2}$)	Mocap	Imu
m1r1t1	0.98	5.32	7.40	0.66	10.83	10.73	0.82	16.19	15.40
m2r1t2	0.63	2.41	3.64	1.43	10.33	11.87	0.91	13.53	14.07
m1r2t1	0.61	6.54	6.77	0.77	12.85	12.88	0.72	15.57	15.83
m2r2t2	1.00	4.73	5.04	1.03	12.71	13.61	0.83	17.74	17.82
m1r3t1a	0.49	8.23	8.15	0.89	14.05	10.02	0.33	8.65	9.45
m2r3t2a	0.99	7.45	7.13	0.98	14.79	9.06	0.72	11.09	11.11
m1r3t1b	3.46	22.72	13.05	1.34	13.75	13.07	1.59	10.85	13.08
m2r3t2b	2.27	11.69	10.76	2.33	9.87	12.15	1.98	15.98	13.22
m1r3t1c	1.73	14.30	6.96	1.17	10.09	11.9	1.05	7.37	6.11
m2r3t2c	1.55	11.28	11.69	2.19	12.14	17.26	1.35	13.43	11.65

**Table 5:** Covariances (per axis) between mocap positional data and double integrated IMU data—selected takes from musicians 1 and 2.

Axis	x		y		z	
Take	x-Covar	Lag	x-Covar	Lag	x-Covar	Lag
(stronger lobe)						
m1r1t2	0.605	−2	0.820	−3	0.865	−3
m2r1t1	0.169	−12	0.841	−1	0.844	0
m1r2t2	0.116	−1	0.603	0	0.673	0
m2r2t1	0.107	−5	0.843	−1	0.840	−1
(weaker lobe)						
m1r1t2	0.313	−4	0.798	−3	0.875	−3
m2r1t1	0.258	−12	0.807	−1	0.872	0
m1r2t2	0.451	0	0.526	−1	0.672	0
m2r2t1	0.138	−6	0.777	−1	0.856	−1

**Table 6:** RMSE and maximal ranges (per axis and method) in the comparison of displacement data of both systems—selected takes from musicians 1 and 2.

Axis	x			y			z		
	RMSE	ΔMax		RMSE	ΔMax		RMSE	ΔMax	
take	(cm)	Mocap	Imu	(cm)	Mocap	Imu	(cm)	Mocap	Imu
(stronger lobe)									
m1r1t2	0.93	1.842	2.64	1.62	3.48	6.55	2.56	6.96	9.75
m2r1t1	0.67	0.882	3.43	1.94	4.75	7.49	1.29	7.94	8.86
m1r2t2	3.23	2.926	16.56	2.89	5.86	15.90	2.62	8.28	16.76
m2r2t1	1.96	2.210	9.11	1.31	5.13	8.91	3.08	9.17	10.67
(weaker lobe)									
m1r1t2	0.89	1.842	1.87	1.52	3.48	2.81	2.48	6.96	5.81
m2r1t1	0.26	0.882	0.98	1.44	4.75	3.97	1.11	7.94	5.58
m1r2t2	1.21	2.926	2.55	2.37	5.86	3.18	1.96	8.28	5.90
m2r2t1	1.20	2.210	1.68	0.95	5.13	3.78	3.13	9.17	6.12

**Table 7:** Covariances between displacement magnitude of each system with two different rotation matrices and two integration methods—musicians 1 and 2.

Take	Mocap-Rotated		IMU-Rotated	
	(s. lobe)	(w. lobe)	(s. lobe)	(w. lobe)
m1r1t1	0.868	0.918	0.863	0.902
m1r1t2	0.868	0.882	0.807	0.804
m1r2t1	0.622	0.705	0.592	0.729
m1r2t2	0.558	0.685	0.174	0.722
m2r1t1	0.834	0.871	0.675	0.747
m2r1t2	0.814	0.836	0.656	0.733
m2r2t1	0.766	0.846	0.765	0.848
m2r2t2	0.886	0.886	0.759	0.882

## Supplementary Materials

The following are available online at https://www.mdpi.com/1424-8220/20/19/5722/s1. Tables S1–S12: All results of the data comparison. Figure S1: Histogram of timestamps of two sensors with a bin width of 15 ms. Figure S2: Process of integrating the acceleration by partially overlapping cycles. Figure S3: Displacement curves estimated for each axis of take m1r2t2, balanced according to the weaker lobe.
