# Supplementary material for: Evaluation of Inertial Sensor Data by a Comparison with Optical Motion Capture Data of Guitar Strumming Gestures
Source: Sensors (Basel). 2020 Oct 8;20(19):5722. doi: 10.3390/s20195722 (PMC7583031; doi:10.3390/s20195722)
Supplement: Supplementary file 1 [file sensors-20-05722-s001.pdf]

Supplementary Materials Related to the Article "Evaluation of Inertial Sensor Data by a Comparison with Optical Motion Capture Data of Guitar Strumming Gestures"

Sérgio Freire, Geise Santos, Augusto Armondes, Eduardo Menseses, Marcelo Wanderley

August 2020

1 Supplementary Figures

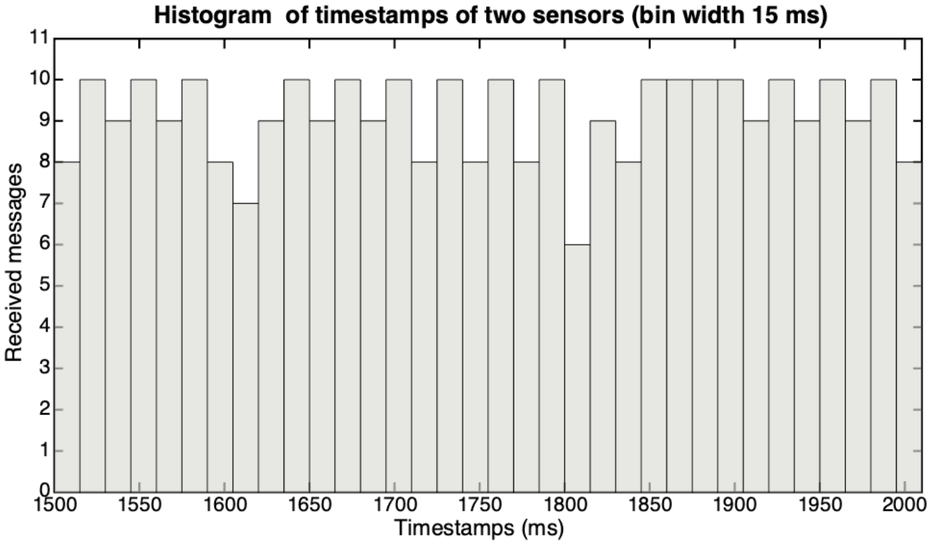

Figure S1: Histogram of timestamps of two sensors with a bin width of 15 ms.

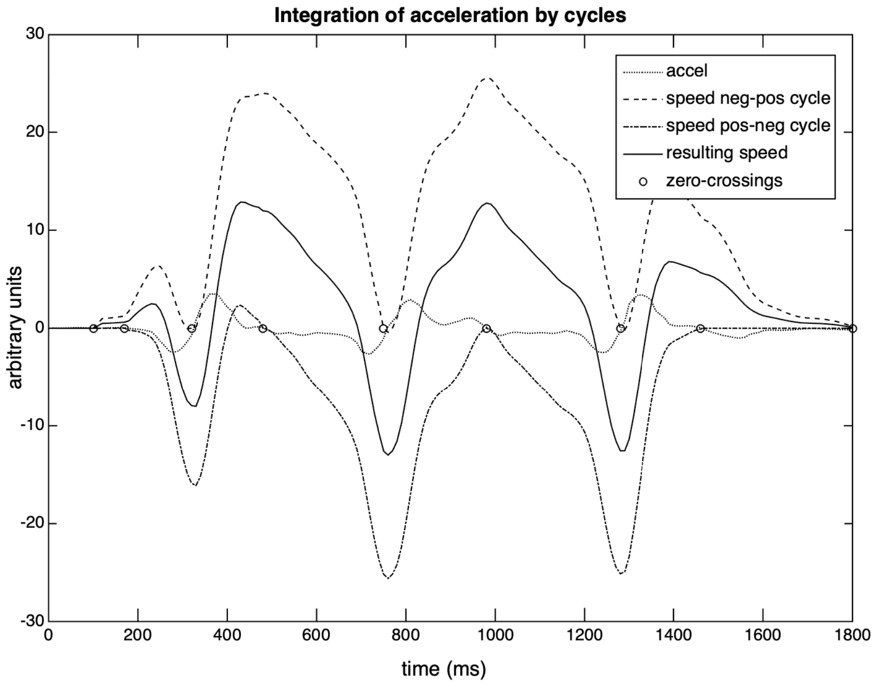

Figure S2: Process of integrating the acceleration by partially overlapping cycles.

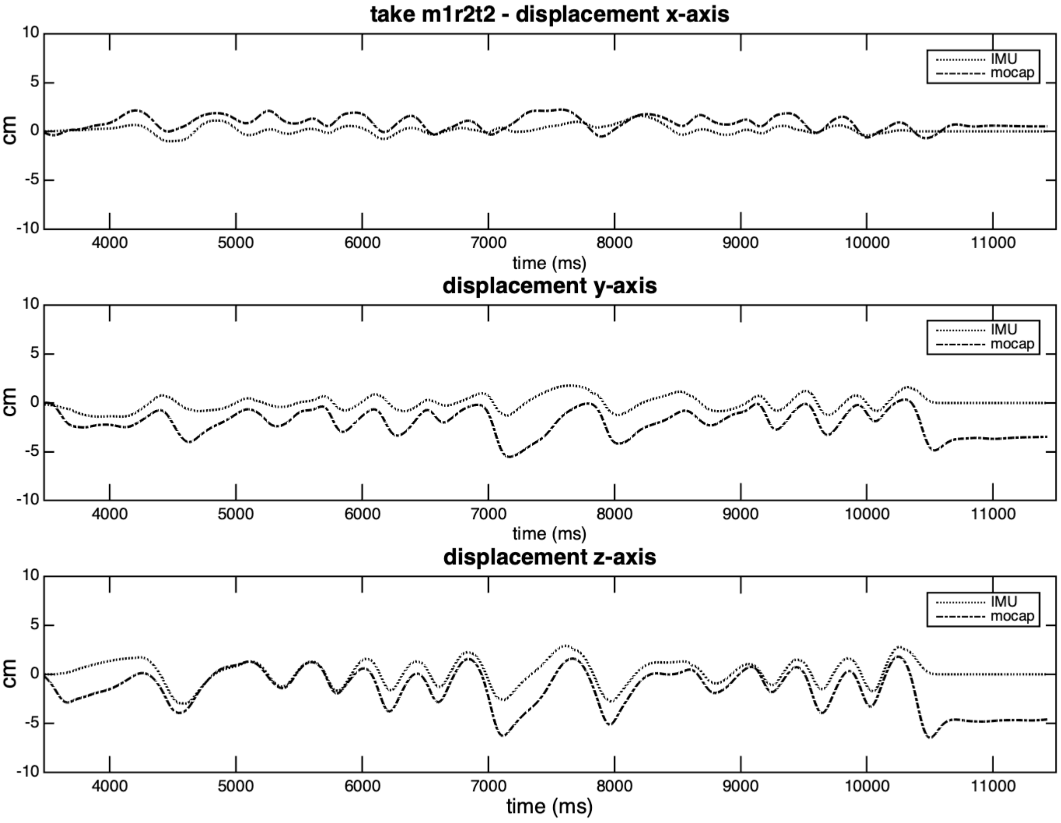

Figure S3: Displacement curves estimated for each axis of take **m1r2t2**, balanced according to the weaker lobe.

2 Data Comparison Tables

2.1 Comparison of Rotational Data

Tables S1 and S2.

Table S1: RMSE and covariances (per axis) between rotational data generated by each system—Musician 1.

take	axis	RMSE (°)	lag	x-covar	mocap mean (°)	stdev	imu mean (°)	stdev
**m1r1t1**	x	4.42	0	0.997	80.7	17.4	76.5	17.4
	y	0.68	0	0.993	29.1	5.8	29.1	5.7
	z	3.48	-8	0.877	-293.8	6.6	-293.7	5.5
**m1r1t2**	x	3.97	3	0.989	82.0	19.1	78.5	18.8
	y	1.16	3	0.990	30.3	7.2	29.8	7.0
	z	2.90	-3	0.929	-290.1	8.0	-289.7	6.8
**m1r2t1**	x	3.24	0	0.993	83.3	17.3	80.9	17.9
	y	1.33	0	0.991	38.6	10.1	38.5	10.1
	z	6.25	-4	0.820	74.4	10.1	73.4	10.0
**m1r2t2**	x	3.94	0	0.975	89.1	15.3	90.6	16.3
	y	3.28	0	0.989	36.1	8.8	39.1	9.0
	z	6.54	-4	0.744	75.6	8.5	75.5	9.4
**m1r3t1a**	x	4.43	-1	0.972	-68.3	3.9	-72.5	4.4
	y	2.36	0/-1	0.994	-23.9	7.7	-26.0	7.7
	z	14.47	-1	0.839	-119.7	9.6	-106.9	3.7
**m1r3t2a**	x	4.27	0	0.966	-76.7	4.8	-80.7	5.5
	y	2.91	0	0.978	-17.9	6.1	-20.5	5.9
	z	14.78	-1	0.898	-123.9	8.2	-110.3	2.9
**m1r3t1b**	x	7.87	0/-1	0.989	-73.6	9.4	-81.2	8.2
	y	4.64	-1	0.866	-4.0	1.6	0.6	0.9
	z	2.74	-1	0.987	-96.8	9.6	-94.8	9.8
**m1r3t2b**	x	5.20	0	0.994	-75.4	9.7	-80.0	11.9
	y	11.87	-1	0.924	-4.8	1.4	-16.7	1.2
	z	2.95	0	0.989	-98.9	11.0	-97.5	8.9
**m1r3t1c**	x	11.09	-1	0.990	-76.8	8.0	-87.8	7.7
	y	1.56	-1	0.990	-1.1	4.1	-2.5	4.0
	z	6.83	-2	0.505	-93.5	4.7	-88.0	2.7
**m1r3t2c**	x	7.82	-1	0.986	-79.9	9.7	-87.5	9.0
	y	1.28	0	0.992	0.9	5.5	-0.2	5.4
	z	7.82	-12	0.611	-96.8	7.2	-91.6	2.8

Table S2: RMSE and covariances (per axis) between rotational data generated by each system—Musician 2.

take	axis	RMSE (°)	lag	x-covar	mocap mean (°)	stdev	imu mean (°)	stdev
**m2r1t1**	x	5.00	1/0	0.996	-80.4	19.4	-85.1	19.4
	y	1.19	0	0.995	-41.4	9.1	-40.7	9.6
	z	4.45	-2	0.969	213.8	13.5	214.3	10.2
**m2r1t2**	x	4.22	2	0.996	-78.1	19.7	-82.2	19.7
	y	1.14	2	0.996	-38.4	8.5	-39.1	8.9
	z	4.37	0	0.974	217.0	13.4	218.7	10.5
**m2r2t1**	x	6.07	1	0.996	-85.1	14.1	-91.2	13.6
	y	0.79	1	0.997	-44.1	9.8	-44.2	10.0
	z	8.35	-1	0.880	209.9	11.0	205.1	12.3
**m2r2t2**	x	6.69	-1	0.996	-79.7	14.7	-86.2	14.2
	y	0.79	-1	0.996	-42.3	9.3	-42.4	9.6
	z	5.13	-3	0.890	212.8	10.8	212.3	11.0
**m2r3t1a**	x	5.63	1/2	0.950	-64.6	5.7	-69.8	5.7
	y	2.83	2	0.972	-27.7	8.9	-29.5	9.0
	z	9.72	6/5	0.711	-150.5	9.3	-143.1	6.9
**m2r3t2a**	x	5.68	0/1	0.948	-67.8	5.8	-73.1	6.1
	y	2.54	1	0.958	-30.6	7.9	-31.4	8.5
	z	11.20	2/1	0.730	-150.1	7.6	-140.2	6.6
**m2r3t1b**	x	3.73	2	0.970	-68.7	6.6	-72.3	6.9
	y	2.48	2	0.979	-11.4	6.4	-9.2	6.3
	z	11.02	4/3	0.914	-122.5	9.9	-113.2	4.3
**m2r3t2b**	x	3.29	0	0.985	-61.4	7.7	-63.6	9.6
	y	7.60	0	0.994	-8.7	9.6	-16.3	9.8
	z	13.39	2/1	0.928	-131.8	11.5	-121.6	3.1
**m2r3t1c**	x	4.37	3	0.988	-71.5	11.8	-75.5	11.9
	y	2.69	3	0.966	-5.0	5.0	-2.6	5.2
	z	5.17	3	0.662	-116.7	5.8	-113.9	3.3
**m2r3t2c**	x	4.40	1	0.987	-64.4	11.0	-68.4	11.4
	y	7.64	1	0.993	-5.3	7.1	-12.9	7.4
	z	8.62	0	0.580	-121.6	6.9	-115.2	5.4

2.2 Comparison of Translations

2.2.1 Deriving the Positional Data

Tables S3, S4, S5 and S6.

Table S3: Covariances (per axis) between linear accelerations from IMU and double derivatives of mocap positional data—Musician 1.

axis	x		y		z	
**take**	**x-covar**	**lag**	**x-covar**	**lag**	**x-covar**	**lag**
**m1r1t1**	0.808	1	0.948	0	0.978	-1
**m1r1t2**	0.709	-2	0.895	-3	0.967	-3
**m1r2t1**	0.854	0	0.948	-1	0.968	-1
**m1r2t2**	0.843	0	0.955	0	0.955	0
**m1r3t1a**	0.952	0	0.899	1	0.973	0
**m1r3t1b**	0.809	0	0.970	0	0.936	1
**m1r3t1c**	0.719	-1	0.788	0	0.627	1
**m1r3t2a**	0.973	-1	0.919	0	0.975	0
**m1r3t2b**	0.911	-1	0.934	0	0.911	0
**m1r3t2c**	0.677	-3	0.768	0	0.638	1

Table S4: RMSE and maximal ranges (per axis) in the comparison of acceleration data of both systems—Musician 1.

axis	x			y			z		
	**RMSE**	**Δmax**		**RMSE**	**Δmax**		**RMSE**	**Δmax**	
**take**	(m/s^2)	**mocap**	**imu**	(m/s^2)	**mocap**	**imu**	(m/s^2)	**mocap**	**imu**
**m1r1t1**	0.98	5.32	7.40	0.66	10.83	10.73	0.82	16.19	15.40
**m1r1t2**	1.30	5.94	8.23	0.98	9.71	9.46	0.96	15.73	15.51
**m1r2t1**	0.61	6.54	6.77	0.77	12.85	12.88	0.72	15.57	15.83
**m1r2t2**	1.02	6.26	9.43	0.62	12.59	14.04	0.91	15.15	17.24
**m1r3t1a**	0.49	8.23	8.15	0.89	14.05	10.02	0.33	8.65	9.45
**m1r3t1b**	3.46	22.72	13.05	1.34	13.75	13.07	1.59	10.85	13.08
**m1r3t1c**	1.73	14.30	6.96	1.17	10.09	11.9	1.05	7.37	6.11
**m1r3t2a**	0.65	9.21	6.80	0.86	14.64	10.64	0.40	8.99	10.77
**m1r3t2b**	2.73	24.70	13.31	2.27	14.70	21.89	1.06	9.89	9.81
**m1r3t2c**	1.82	14.24	6.45	1.16	10.41	9.76	1.17	8.75	7.35

Table S5: Covariances (per axis) between linear accelerations from IMU and double derivatives of mocap positional data—Musician 2.

axis	x		y		z	
**take**	**x-covar**	**lag**	**x-covar**	**lag**	**x-covar**	**lag**
**m2r1t1**	0.478	-3	0.950	0	0.979	0
**m2r1t2**	0.549	-3	0.876	-2	0.970	-2
**m2r2t1**	0.246	-2	0.932	0	0.980	-1
**m2r2t2**	0.447	0	0.956	1	0.989	1
**m2r3t1a**	0.929	-3	0.690	-3	0.985	-3
**m2r3t1b**	0.806	-4	0.402	-4	0.929	-3
**m2r3t1c**	0.743	-4	0.730	-4	0.763	-3
**m2r3t2a**	0.787	-2	0.930	-2	0.955	-2
**m2r3t2b**	0.679	-1	0.658	-1	0.848	-1
**m2r3t2c**	0.753	-1	0.738	-1	0.837	-1

Table S6: RMSE and maximal ranges (per axis) in the comparison of acceleration data of both systems—Musician 2.

**axis**	**x**			**y**			**z**		
	**RMSE**	**Δmax**		**RMSE**	**Δmax**		**RMSE**	**Δmax**	
**take**	(m/s^2)	**mocap**	**imu**	(m/s^2)	**mocap**	**imu**	(m/s^2)	**mocap**	**imu**
**m2r1t1**	0.58	2.61	3.27	0.99	10.83	12.76	0.85	12.78	11.65
**m2r1t2**	0.63	2.41	3.64	1.43	10.33	11.87	0.91	13.53	14.07
**m2r2t1**	1.58	5.22	7.26	1.42	13.06	13.97	1.24	19.09	17.19
**m2r2t2**	1.00	4.73	5.04	1.03	12.71	13.61	0.83	17.74	17.82
**m2r3t1a**	0.90	9.77	11.08	1.74	15.38	8.48	0.61	13.26	10.99
**m2r3t1b**	2.03	12.61	8.73	2.13	10.55	4.51	1.23	13.34	13.13
**m2r3t1c**	1.76	12.70	10.85	1.78	15.26	15.81	1.35	10.76	10.03
**m2r3t2a**	0.99	7.45	7.13	0.98	14.79	9.06	0.72	11.09	11.11
**m2r3t2b**	2.27	11.69	10.76	2.33	9.87	12.15	1.98	15.98	13.22
**m2r3t2c**	1.55	11.28	11.69	2.19	12.14	17.26	1.35	13.43	11.65

2.2.2 Integrating the Acceleration Data

Tables S7, S8, S9 and S10 for individual axes. Tables S11 and S12 for moduli.

Table S7: Covariances (per axis) between mocap positional data and double integrated IMU data—Musician 1.

**axis**	**x**		**y**		**z**	
**take**	**x-covar**	**lag**	**x-covar**	**lag**	**x-covar**	**lag**
			(stronger lobe)			
**m1r1t1**	0.306	-4	0.833	-1	0.897	-1
**m1r1t2**	0.605	-2	0.820	-3	0.865	-3
**m1r2t1**	0.499	0	0.515	-1	0.547	-1
**m1r2t2**	0.116	-1	0.603	0	0.673	0
			(weaker lobe)			
**m1r1t1**	0.316	-2	0.841	0	0.925	-1
**m1r1t2**	0.313	-4	0.798	-3	0.875	-3
**m1r2t1**	0.468	0	0.647	-2	0.653	-1
**m1r2t2**	0.451	0	0.526	-1	0.672	0

Table S8: Covariances (per axis) between mocap positional data and double integrated IMU data—Musician 2.

**axis**	**x**		**y**		**z**	
**take**	**x-covar**	**lag**	**x-covar**	**lag**	**x-covar**	**lag**
			(stronger lobe)			
**m2r1t1**	0.169	-12	0.841	-1	0.844	0
**m2r1t2**	0.402	2	0.852	-3	0.829	-2
**m2r2t1**	0.107	-5	0.843	-1	0.840	-1
**m2r2t2**	0.075	-1	0.831	0	0.870	1
			(weaker lobe)			
**m2r1t1**	0.258	-12	0.807	-1	0.872	0
**m2r1t2**	0.506	6	0.781	-3	0.837	-2
**m2r2t1**	0.138	-6	0.777	-1	0.856	-1
**m2r2t2**	0.213	-1	0.819	1	0.889	1

Table S9: RMSE and maximal ranges (per axis and method) in the comparison of displacement data of both systems—Musician 1.

**axis**		**x**			**y**			**z**	
	**RMSE**	**Δmax**		**RMSE**	**Δmax**		**RMSE**	**Δmax**	
**take**	(cm)	**mocap**	**imu**	(cm)	**mocap**	**imu**	(cm)	**mocap**	**imu**
				**(stronger lobe)**					
**m1r1t1**	1.11	1.751	6.19	0.67	2.96	4.20	1.79	7.26	9.00
**m1r1t2**	0.93	1.842	2.64	1.62	3.48	6.55	2.56	6.96	9.75
**m1r2t1**	0.85	2.528	3.13	2.23	5.93	8.87	2.43	8.73	18.10
**m1r2t2**	3.23	2.926	16.56	2.89	5.86	15.90	2.62	8.28	16.76
				**(weaker lobe)**					
**m1r1t1**	0.52	1.751	1.28	0.54	2.96	2.52	1.54	7.26	5.13
**m1r1t2**	0.89	1.842	1.87	1.52	3.48	2.81	2.48	6.96	5.81
**m1r2t1**	0.81	2.528	1.43	1.62	5.93	3.21	1.66	8.73	5.30
**m1r2t2**	1.21	2.926	2.55	2.37	5.86	3.18	1.96	8.28	5.90

Table S10: RMSE and maximal ranges (per axis and method) in the comparison of displacement data of both systems—Musician 2.

**axis**	**x**			**y**			**z**		
	**RMSE**	**Δmax**		**RMSE**	**Δmax**		**RMSE**	**Δmax**	
**take**	(*cm*)	**mocap**	**imu**	(*cm*)	**mocap**	**imu**	(*cm*)	**mocap**	**imu**
				**(stronger lobe)**					
**m2r1t1**	0.67	0.882	3.43	1.94	4.75	7.49	1.29	7.94	8.86
**m2r1t2**	0.62	0.872	2.68	1.45	4.84	8.22	1.43	8.07	9.47
**m2r2t1**	1.96	2.210	9.11	1.31	5.13	8.91	3.08	9.17	10.67
**m2r2t2**	0.62	2.172	2.71	2.58	5.35	6.66	1.34	8.42	8.66
				**(weaker lobe)**					
**m2r1t1**	0.26	0.882	0.98	1.44	4.75	3.97	1.11	7.94	5.58
**m2r1t2**	0.23	0.872	1.20	0.98	4.84	3.47	1.44	8.07	6.26
**m2r2t1**	1.20	2.210	1.68	0.95	5.13	3.78	3.13	9.17	6.12
**m2r2t2**	0.47	2.172	1.15	2.44	5.35	3.78	1.32	8.42	6.23

Table S11: Covariances between displacement magnitude of each system with two different rotation matrices and two integration methods—musicians 1 and 2.

**take**	**mocap-rotated**		**IMU-rotated**	
	(s. lobe)	(w. lobe)	(s. lobe)	(w. lobe)
**m1r1t1**	0.868	0.918	0.863	0.902
**m1r1t2**	0.868	0.882	0.807	0.804
**m1r2t1**	0.622	0.705	0.592	0.729
**m1r2t2**	0.558	0.685	0.174	0.722
**m2r1t1**	0.834	0.871	0.675	0.747
**m2r1t2**	0.814	0.836	0.656	0.733
**m2r2t1**	0.766	0.846	0.765	0.848
**m2r2t2**	0.886	0.886	0.759	0.882

Table S12: RMSE and maximal ranges in the comparison of displacement moduli of each system with two different rotation matrices and two integration methods—musicians 1 and 2.

**take**	**RMSE** (cm)		**Δmax** (cm)		
	**(s. lobe)**	**(w. lobe)**	**mocap**	**(s. lobe)**	**(w. lobe)**
			(mocap-rotated)		
**m1r1t1**	3.80	0.74	6.19	9.90	4.77
**m1r1t2**	3.86	0.99	6.09	10.25	5.34
**m1r2t1**	7.55	1.14	6.46	16.81	5.08
**m1r2t2**	11.65	1.24	6.85	19.01	6.01
**m2r1t1**	3.77	1.18	5.72	9.91	5.72
**m2r1t2**	4.73	1.51	5.92	10.96	5.41
**m2r2t1**	6.95	1.19	7.09	13.64	6.49
**m2r2t2**	3.33	1.15	6.40	9.43	6.56
			(imu-rotated)		
**m1r1t1**	3.04	0.93	6.19	6.26	3.45
**m1r1t2**	3.34	1.22	6.09	6.83	4.38
**m1r2t1**	10.02	1.19	6.46	22.07	4.64
**m1r2t2**	17.43	1.34	6.85	25.45	4.12
**m2r1t1**	2.84	1.49	5.72	4.95	3.34
**m2r1t2**	3.07	1.61	5.92	3.98	3.49
**m2r2t1**	3.07	1.61	7.09	5.07	4.12
**m2r2t2**	2.66	1.40	6.40	4.43	4.07
